# Prevalence and Severity of Dry Eye Disease Symptoms Among Diabetics: A Nationwide Survey

**DOI:** 10.7759/cureus.30981

**Published:** 2022-11-01

**Authors:** Baqer A Almohammed, Aryaf A Alnafeesah, Sarah S Aldharman, Manal H Alenzi, Ahood A Mahjari, Faisal A Albalawi, Khaled A Amer, Ghaythah H Alkhathami, Abdulaziz A Al Taisan

**Affiliations:** 1 Medicine, Al-Jabr Eye and Ear, Nose, Throat (ENT) Hospital, Al-Ahsa, SAU; 2 College of Medicine, Qassim University, Unaizah, SAU; 3 College of Medicine, King Saud Bin Abdulaziz University for Health Sciences, Riyadh, SAU; 4 College of Medicine, Northern Border University, Arar, SAU; 5 College of Medicine, Najran University, Najran, SAU; 6 College of Medicine, Jouf University, Al-Jouf, SAU; 7 College of Medicine, King Khalid University, Abha, SAU; 8 College of Medicine, King Abdulaziz University, Jeddah, SAU; 9 Collage of Medicine, King Faisal University, Al-Ahsa, SAU

**Keywords:** dry eye disease, ocular surface disease index, diabetes mellitus, hba1c, saudi arabia, corneal sensitivity

## Abstract

Introduction: Diabetes mellitus (DM) microvascular complications can impair corneal sensitivity and lacrimal gland functioning, leading to dry eye disease (DED). Hence, this study aimed to measure the prevalence and severity of DED symptoms, and the related risk factors, among the Saudi diabetic population.

Methods: This is a retrospective, cross-sectional, survey-based study which targeted Saudi adults (20 years and older) previously diagnosed with type 1 or type 2 DM. It was conducted in eight primary healthcare centers (PCHs) scattered around eight different provinces of Saudi Arabia (SA). The prevalence and severity of DED were measured by the Ocular Surface Disease Index (OSDI).

Results: The total study population was 389 subjects, of which 182 (46.8%) were males and 207 (53.2%) were females. The overall prevalence of DED was 51.7%. Among those, 20.3% of patients had mild dryness, 11.1% had moderate dryness, and 20.3% had severe dryness. Glycosylated hemoglobin (HbA1c) levels of 6.5% or higher proved to be an independent risk factor for the development of DED symptoms, 3.6-folds higher for HbA1c levels of 6.5% to 9% (AOR=3.573; p=0.001), and 2.3-folds higher for HbA1c levels higher than 9% (AOR=2.293; p=0.013). The long duration of diabetes did not show a significant association with manifesting DED symptoms (p=0.263).

Conclusion: Half of the diabetic population complained of DED symptoms, compared to one-third of the previously studied general Saudi population. Patients with mild to moderate HbA1c elevation were more likely to report DED symptoms than those with severe elevation, which could be related to impaired corneal sensation. Therefore, a routine ophthalmological examination is recommended.

## Introduction

Dry eye disease (DED) can be defined as "a multifactorial disease of the ocular surface characterized by a loss of homeostasis of the tear film and accompanied by ocular symptoms in which tear film instability and hyperosmolarity, ocular surface inflammation and damage, and neurosensory abnormalities play etiological roles." [1]. DED is classified as aqueous-deficient when the dryness is caused by reduced tear production, and hyper-evaporative when it is related to increased evaporation of the tear film; however, mixed forms are common [2].

Many risk factors have been implicated in the development of this condition. Some of the high-level evidence risk factors are age, female sex, antihistamines, collagen vascular disease, corneal refractive surgery, and vitamin A deficiency. In addition, there are other moderately evidenced risk factors such as diabetes mellitus (DM), isotretinoin, low air humidity, and human immunodeficiency virus infection [3].

DM is a common metabolic disease in Saudi Arabia (SA); it is estimated to affect around 18.3% of Saudi adults [4]. DM can lead to several ocular complications, one of which is DED [5-7]. Diabetic peripheral neuropathy results in impaired corneal sensitivity and diminished reflex tearing. This factor, coupled with microvascular damage to the lacrimal gland and impairment of the lacrimal gland innervation in diabetic autonomic neuropathy, leads to diminished tear production [6,7].

Symptoms of DED include eye redness, burning, stinging, foreign-body sensation, pruritus, and photophobia [3]. Neglected late stages and severe forms of the disease can cause ocular complications which might be vision-threatening in some cases, these include conjunctival scarring, filamentary keratitis, persistent epithelial defects, ulceration, and even corneal perforation [2].

Although a study found that the overall prevalence of DED symptoms was 32.1% in the Saudi population [8], the literature is deficient regarding the prevalence of this disease among the diabetic population. Furthermore, early diagnosis and treatment of the condition are important to prevent the development of a "vicious circle" of escalating inflammation [9]. As a result, the aim of this study was to estimate the prevalence of DED symptoms, their severity, and the related risk factors among the diabetic population of the Kingdom of Saudi Arabia.

## Materials and methods

Study design

This is a retrospective, cross-sectional, questionnaire-based study that targeted Saudi diabetic adults. Its aim was to investigate the presence of DED symptoms and their severity in the selected study population. The conduct of the study followed the Declaration of Helsinki guidelines.

Setting

The study was conducted in eight ministry of health's (MOH) public primary healthcare centers (PHCs). The survey was distributed between eight provinces covering the east, west, north, south, and central SA. The involved provinces were the Eastern Province, Riyadh, Al-Qassim, Makkah, Northern Borders, Al-Jouf, Asir, and Najran.

Study population

The Saudi adult population (20 years and older) is estimated to be around 12,649,150 inhabitants [10]. The estimated prevalence of DM in Saudi adults (20 years and older) is 18.3%, which approximately equals 2,314,794 from the previously mentioned population [4].

The population of interest that was included is all Saudi adults (20 years and above) who were previously diagnosed with type 1 or type 2 DM by a healthcare professional, who presented to the assigned MOH PHCs and agreed to participate in the study by signing the consent form. The exclusion criteria were: age less than 20 years, a history of eye surgery within the last month, active ocular infection, recent ocular trauma, and critical illness.

Sampling

A minimum sample size of 224 participants was required. It was calculated using Epi‑Info™ software, version 7.2. It was based on a population size of 2,314,794 individuals, a 95% confidence interval, 5% marginal error, and 17.7% expected frequency based on a previous similar study, which utilized the ocular surface disease index (OSDI) [11]. The total number of participants was increased to 389 subjects to increase the statistical power. A convenience sampling technique was adopted to recruit the eligible participants.

Study procedure

The outcome of this study was measured by the OSDI, which is a 12-item questionnaire whose validity and reliability have been tested and confirmed for assessment of the DED symptoms, their severity, and their impact on vision-related functioning [12,13].

The original questionnaire was translated into Arabic by an expert. A pilot study was conducted on 15 random subjects to evaluate the clarity, ease of understanding, and convenience. The questionnaire was handed to the chosen participants to be filled out after they signed a consent sheet that stated the nature and purpose of the study.

The first part of the survey included questions about sociodemographic characteristics including age, gender, residency, level of education, and smoking.

The second part asked DM-related questions, including the type of DM, duration since diagnosis, last known glycosylated hemoglobin (HbA1c) result, and the treatment, whether oral medications, insulin injections, or both [14].

The third part was the OSDI, which contained 12 questions assessing the DED symptoms resulting from ocular irritation in addition to their influence on vision-related functioning in the previous seven days [12,13]. Answers to each question were given a score from 0 to 4, in which 0 represents none of the time, 1 some of the time, 2 half of the time, 3 most of the time, and 4 all the time. The final OSDI scores of 0-12.99 were considered normal, 13-22.99 mild, 23-32.99 moderate, and 33-100 severe disease [15]. The final score was calculated by the following formula:

OSDI = sum of scores for all answered questions*25/number of the answered questions

The primary outcome investigated was to assess if diabetic patients have a high prevalence of DED. The secondary outcomes were to measure if the prevalence of DED in diabetics was higher than in non-diabetics investigated in other studies. In addition, the correlation between the DED and the length of duration since DM diagnosis and the level of disease control were investigated. The study was conducted during the period from January 2022 to June 2022.

Data analysis and management

Categorical variables were presented as numbers and percentages (%), while continuous variables were summarized as mean and standard deviation. The relationship between the level of DED and the socio-demographic characteristics of diabetic patients has been tested using the Chi-square test. Significant results were then placed into a multivariate regression model to determine the independent significant factor associated with DED. Two-tailed analyses with p<0.05 were used as the cutoff for statistical significance. All data analyses were performed using the statistical package for social sciences, version 26 (SPSS, IBM Corp., Armonk, NY, USA). Confidentiality of all obtained data from the patients is maintained as there was no collection of any identifiable information from any participant.

## Results

**Figure 1:**
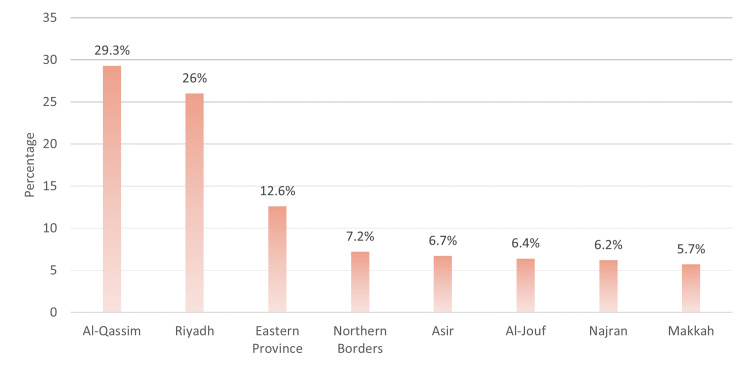
Distribution of study subjects across the different provinces of Saudi Arabia (n=389).

Out of 465 participants in this study, 389 diabetic patients fit the inclusion and exclusion criteria. As seen in Table 1, the most common age group was 56-65 years old (25.4%), with more than half (53.2%) being females. Patients mostly lived in the city (83.8%). Patients who were university degree graduates constituted 58.1% of the total study population. The prevalence of cigarette smoking was 17.7%, while the proportion of patients who were using artificial tears was 40.9%. Patients with type 2 diabetes were 64.8% and patients with a duration of 6-10 years were 26.7%. The most commonly used treatment method was oral medications (50.1%), while 41.9% were on insulin injections only. Additionally, a little below half of them (48.8%) had last HbA1c results of 6.5% to 9%. Figure 1 shows the province of residence of the patients. It can be observed that 29.3% were living in Al-Qassim province and 26% were living in Riyadh province.

**Table 1:** Sociodemographic and diabetes mellitus characteristics (n=389).

Factor	Categories	Frequency	Percentage
Age group	20–25 years	81	20.8%
	26–35 years	57	14.7%
	36–45 years	39	10.0%
	46–55 years	83	21.3%
	56–65 years	99	25.4%
	>65 years	30	07.7%
Gender	Male	182	46.8%
	Female	207	53.2%
Residence	City	326	83.8%
	Village	63	16.2%
Level of education	No formal education	20	05.1%
	Elementary school	38	09.8%
	Intermediate school	30	07.7%
	High school	75	19.3%
	University	226	58.1%
Smoking	Non-smoker	320	82.3%
	Smoker	69	17.7%
Use of artificial tears/eye lubricants	Yes	159	40.9%
	No	230	59.1%
Type of diabetes	Type 1	137	35.2%
	Type 2	252	64.8%
Duration of diabetes	0–6 months	38	09.8%
	7 months to 1 year	23	05.9%
	2–5 years	73	18.8%
	6–10 years	104	26.7%
	11–20 years	100	25.7%
	>20 years	51	13.1%
Treatment type	Insulin injections	163	41.9%
	Oral medications	195	50.1%
	Both	31	08.0%
Last HbA1c results	<6.5%	83	21.3%
	6.5–9.0%	190	48.8%
	>9.0%	56	14.4%
	Unknown	60	15.4%

The prevalence of DED is demonstrated in Table 2. It was revealed that the mean OSDI score was 19.6 (SD 18.4). According to the given criteria, the prevalence of patients with DED was 51.7%, and the rest did not suffer from DED (48.3%). Table 2 shows that 20.3% of the people had mild DED, 11.1% had moderate DED, and 20.3% had severe DED.

**Table 2:** Prevalence of dry eye disease according to OSDI questionnaire (n=389). OSDI: Ocular Surface Disease Index.

OSDI parameters	Frequency	Percentage
OSDI score (mean ± SD)	19.6 ± 18.4	
Level of DED		
Have DED (score 13–100)	201	51.7%
No DED (score 0–12.99)	188	48.3%
Severity of DED		
Normal (score 0–12.99)	188	48.3%
Mild (score 13–22.99)	79	20.3%
Moderate (score 23–32.99)	43	11.1%
Severe (score 33–100)	79	20.3%

When measuring the relationship between DED and the socio-demographic characteristics of the patients (Table 3), it was found that the prevalence of patients with DED was significantly higher among the older age groups who were older than 45 years (p<0.001), those who used artificial tears (p 0.001), those diagnosed with type 2 diabetes (p=0.003), and those who had last HbA1c results of more than 9% (p=0.045). Other variables such as gender, residence location, level of education, smoking status, duration of diabetes, and treatment type did not show a significant relationship with DED (p>0.05).

**Table 3:** Relationship between dry eye disease and the socio-demographic factors of the diabetes mellitus patients (n=389). *Significant at p<0.05 level. ^†^P-value has been calculated using the Chi-square test. ^‡^Patients who did not know their last HbA1c results were excluded from the analysis.

Factor	Categories	Level of dry eye disease		P-value^†^
		DED N (%) ^(n=201)^	No DED N (%) ^(n=188)^	
Age group	≤45 years	74 (41.8%)	103 (58.2%)	<0.001*
	>45 years	127 (59.9%)	85 (40.1%)	
Gender	Male	85 (46.7%)	97 (53.3%)	0.066
	Female	116 (56.0%)	91 (44.0%)	
Residence location	City	174 (53.4%)	152 (46.6%)	0.126
	Village	27 (42.9%)	36 (57.1%)	
Level of education	High school or below	90 (55.2%)	73 (44.8%)	0.235
	University degree	111 (49.1%)	115 (50.9%)	
Smoking status	Non-smoker	168 (52.5%)	152 (47.5%)	0.481
	Smoker	33 (47.8%)	36 (52.2%)	
Use of artificial tears	Yes	108 (67.9%)	51 (32.1%)	<0.001*
	No	93 (40.4%)	137 (59.6%)	
Type of diabetes	Type 1	57 (41.6%)	80 (58.4%)	0.003*
	Type 2	144 (57.1%)	108 (42.9%)	
Duration of diabetes	≤5 years	64 (47.8%)	70 (52.2%)	0.263
	>5 years	137 (53.7%)	118 (46.3%)	
Treatment type	Insulin injections	77 (47.2%)	86 (52.8%)	0.170
	Oral medications	104 (53.3%)	91 (46.7%)	
	Both	20 (64.5%)	11 (35.5%)	
Last HbA1c results^‡^	<6.5%	36 (43.4%)	47 (56.6%)	0.045*
	6.5–9.0%	92 (48.4%)	98 (51.6%)	
	>9.0%	36 (64.3%)	20 (35.7%)	

When conducting a multivariate regression model (Table 4), it can be observed that compared to patients who were not using artificial tears, patients who were using artificial tears were predicted to increase the chance of having DED by at least 3 times higher (AOR=3.061; 95% CI=1.856-5.046; p<0.001). In addition, compared to patients who had controlled HbA1c (HbA1c<6.5%), patients who reported having 6.5% to 9% last HbA1c results were predicted to have an increased chance of having DED by at least 3.6-fold higher (AOR=3.573; 95% CI=1.658-7.700; p=0.001) or 2.3-fold higher for patients with HbA1c more than 9% (AOR=2.293; 95% CI=1.188-4.424; p=0.013). Other variables included in the model did not show a significant effect on DED after adjustments to a regression model including age group and type of diabetes.

**Table 4:** Multivariate regression analysis to determine the independent significant factor of dry eye disease (n=389). AOR: adjusted odds ratio; CI: confidence interval. *Significant at p<0.05 level. ^†^Patients who did not know their last HbA1c results were excluded from the analysis.

Factor	Categories	AOR	95% CI	P-value
Age group	≤45 years	Ref		
	>45 years	1.398	0.757–2.584	0.284
Use of artificial tears	No	Ref		
	Yes	3.061	1.856–5.046	<0.001*
Type of diabetes	Type 1	Ref		
	Type 2	1.404	0.757–2.604	0.282
Last HbA1c results^†^	<6.5%	Ref		
	6.5–9.0%	3.573	1.658–7.700	0.001*
	>9.0%	2.293	1.188–4.424	0.013*

## Discussion

To the best of our knowledge, this is the first study to assess DED symptoms among type 1 and type 2 diabetics from multiple provinces of SA. In this study, the overall prevalence of DED amid diabetics, assessed by the OSDI, was 51.7%. The mean and SD were 19.6 ± 18.4. Of the total population in the current study, 20.3% had severe DED. These findings are similar to what Fuerst et al. has reported (52%) [16], with an OSDI score mean and SD of 19.3±18.2, with 18% of his study’s total population having a severe disease. In contrast, the prevalence of DED was found to be lower in a Nigerian population (21.7%), with only 2.1% having a severe disease [14]. However, only 43.9% of the Nigerian patients had an HbA1c ≥6.5%, while the mean was 7%, compared to at least 63.2% of the Saudi patients in this study. Furthermore, the total number of the sample (189) was lower in their study in comparison to this study (389) [14]. When assessing DED by objective tools including tear break up time and Schirmer tests, Manaviat et al. reported a close percentage (54.3%) of the type 2 DM patients having the disease [17].

DED prevalence and severity in the diabetics in our study seem to be high when compared to the rates noted in two other local studies, conducted on the general population in different cities in SA. In a study done by Alshammrani et al. among Al-Ahsa’s general population, the prevalence of DED was 32.1% [8]. Additionally, a study done by Yasir et al. in the Riyadh Governorate’s (except the capital) general population, indicated that 35% of Saudis aged 40 years and older had DED, which ranged from mild to severe as 25%, 9%, and 2%, respectively [18]. However, their study concluded that the magnitude of DED among the Saudi older population was high, but it was of a milder nature to a large extent [18].

On observing the risk factors of DED in DM patients, DED was more prevalent in the age groups older than 45 years (59.9%) and in females (56%). Nevertheless, after adjusting the variables to a multivariate regression model, age and gender did not show an independent significant relationship with DED symptoms. Despite the fact that some studies found that these two factors are associated with DED in the general population [19,20], several studies conducted on diabetic patients exclusively concluded that this association is insignificant [11,14,16,17]. Interestingly, patients who were already using artificial tears were 3 times more likely to report DED symptoms (AOR=3.061; 95% CI=1.856-5.046; p<0.001). Compliance was not measured in this study, as it was observed that regular and frequent use of artificial tears and eye lubricants is essential for optimal control of dryness symptoms [21]. Not all patients respond to artificial tears [22]. Whether diabetic patients are prone to developing a DED refractory to conventional artificial tears is a question for future research.

Regarding DM characteristics, although the duration since DM onset is an important factor for microvascular diabetes complications [23], it did not have a significant relationship with DED symptoms in this study. Several studies have also reported similar results [11,14,16]. In different studies, a significant association was noted between DED assessed by objective tests and a longer duration of DM, such as tear break-up time, Schirmer test, and corneal fluorescein staining scores [17,24]. HbA1c is considered the most important tool for the assessment of DM control. High levels of HbA1c have proved to be related to the development of microvascular complications [23]. In the presented study, HbA1c ≥6.5% was an independent risk factor for the development of DED symptoms. The same result was found in multiple studies [7,11,24]. Last HbA1c level of 6.5-9% was predicted to increase the chance of having DED (AOR=3.573; 95% CI=1.658-7.700; p=0.001) and also HbA1c >9% (AOR=2.293; 95% CI=1.188-4.424; p=0.013). Considering that this study depended on OSDI exclusively, it is notable that OSDI is a subjective tool for the diagnosis of DED, which depends totally on patients’ reporting of their ocular symptoms. Diabetic patients with a long duration of the disease and poor HbA1c control are more likely to have an impaired corneal sensation, as a result, they are less likely to report symptoms of ocular surface irritation [6,7,16]. Galor et al. found that individuals with corneal hyposensitivity exhibit lower scores on subjective assessment of DED symptoms by OSDI and Dry Eye Questionnaire 5, but they show more severe signs of dryness when examined by objective tests including tear break-up time, Schirmer test, and corneal fluorescein staining [25].

After all, the study had some limitations, including a lack of a control group of healthy non-diabetics for comparison. Moreover, it did not combine objective tools for the assessment of DED signs in addition to the assessed symptoms. It is also important to mention that the study did not address the autoimmune conditions that are related to DED as a potential cause of the disease in the study subjects. Specifically, primary or secondary Sjögren's syndrome, which is an autoimmune condition associated with xerostomia in addition to eye dryness [26]. Lastly, HbA1c levels were unknown for 15.4% of the study population; hence, these were excluded from the Chi-square test and the multivariate regression analysis.

## Conclusions

When compared to the general Saudi population, DED among Saudi diabetics was demonstrated to be more prevalent and of a more severe nature. Poor glycemic control, as measured by HbA1c, raises the likelihood of developing DED. Nonetheless, it can also impair corneal sensitivity, hindering the appearance of DED symptoms. In comparison to the symptom-wise approach adopted in this study, eye examination by objective tests adopted by different studies appeared to be more efficient for detecting DED in long-standing and poorly controlled DM. As a result, routine ophthalmological screening for signs of dryness is important in the diabetic population.
